# Home processed complementary foods, caregivers' knowledge, attitude, and practice in a rural community in Kenya: a mixed method study

**DOI:** 10.11604/pamj.2024.49.78.38148

**Published:** 2024-11-14

**Authors:** Susan Moraa Momanyi-Nyasimi, Judith Kimiywe, Hudson Nyambaka

**Affiliations:** 1 Department of Food, Nutrition and Dietetics, Kisii University, Kisii, Kenya,; 2 Department of Food, Nutrition and Dietetics, Kenyatta University, Nairobi, Kenya,; 3 Department of Chemistry, Kenyatta University, Nairobi, Kenya

**Keywords:** Complementary foods, germination, fermentation, young children, western Kenya

## Abstract

**Introduction:**

despite evidence of the health benefits of fermented and germinated foods, consumption is waning, particularly among children in Kenya. We conducted this study to explore the knowledge, attitudes, and use of fermented and germinated complementary foods in Homa-Bay County to advocate for their use as an additional tool to address the prevalent iron and zinc deficiency among children.

**Methods:**

an explorative mixed-study design. Six focus group discussions; followed by a cross-sectional quantitative survey with 314 caregivers. Atlas-ti software and SPSS were used for qualitative and quantitative data analysis.

**Results:**

this community was aware of and practiced germination and fermentation of foods. However, the older caregivers knew 1.5 times more foods than younger caregivers. Caregivers' inadequate knowledge about the benefits of germinated and fermented complementary foods and a negative attitude, prevented children been offered these foods. The caregivers´ greatest concerns were diarrhea following the consumption of fermented food and the potential for stomach ulcer development due to the acidity in these foods. The caregiver's attitude, age, and level of education significantly influenced the use of fermented and germinated complementary foods. A positive attitude (AOR 4.897, 95% CI: 1.616-14.844), being 35 years or older (AOR 2.7, 95% CI: 1.670-4.428), and having no or only a primary level of education (AOR 3.344, 95% CI: 1.319-8.480) were all associated with a higher likelihood of using fermented or germinated foods.

**Conclusion:**

to encourage caregivers to use germinated and fermented complementary foods, there must be concerted efforts to educate the community on the benefits of consuming these foods.

## Introduction

Iron and zinc deficiencies are the most widespread micronutrient deficiencies in Kenya. According to [1], 21.8% and 13.3% of children aged 6-59 months suffered from iron deficiency and iron deficiency anemia (IDA) in Kenya. More recent sub-national studies have confirmed that IDA levels are the same or higher in Kenya [2,3]. Moreover, about 83.3% of children aged under 59 months are zinc deficient in Kenya [1]. Other studies (sub-national) conducted in Kenya have reported high rates of zinc deficiency [3,4]. Iron and zinc deficiencies, if not identified and treated are associated with increased illnesses, slowed growth and development, and death, especially among children under the age of five [5]. There is evidence that the consumption of plant-based staples with high levels of anti-nutrients, inadequate intake of iron and zinc, and low intake of heme-iron-rich animal products are drivers of iron and zinc deficiencies in low- and middle-income settings [6]. Zinc deficiency could also be caused by diseases that impair food intake, cause malabsorption, and increase the excretion of zinc. According to [7,8], the increased demand for zinc during childhood growth if not matched with increased zinc intake may also contribute to zinc deficiency. Given the multiplicity of etiologies for iron and zinc deficiency among children, it is clear that there is no single strategy to eliminate iron and zinc deficiencies in the world [9]. A multipronged approach model combining supplementation, fortification, agricultural interventions, improved food processing, and education to improve dietary quality is all that is necessary [10].

Diets in Kenya and other low-income countries are composed primarily of cereals and legumes that are high in phytates that inhibit zinc and iron absorption [11-13]. The high content of antinutrients could significantly be reduced by the use of fermentation, making a larger share of the micronutrients bioavailable [14]. Moreover, several studies have indicated that fermentation of foods is protective against foodborne diseases, especially diarrhea, which is common when complementary foods are introduced [15-17], and improves the shelf life and nutritional quality of foods and beverages [18,19]. Humans have practiced fermentation of foods for many years [20]. Consumption of fermented food products prepared through traditional fermentation is widespread in developing countries. Unfortunately, the preparation of fermented food products in homes is declining in Kenya [12] contend that changing lifestyles have negatively impacted traditional food consumption, preparation, and storage. The researcher further opines that inadequate transfer of knowledge and skills in the preparation of fermented foods has contributed to the loss of indigenous knowledge and skills in food preparation, food combinations of nutritional value, and food conservation. Although changing people's behavior in terms of how they prepare and process foods is not so easy, it offers hope of a sustainable approach as it enables people to take ultimate responsibility for the quality of their diet [10]. Programmatic experiences in the promotion of home processing of complementary foods are limited [21]. This study´s main objective is to explore the feasibility of utilizing fermented and germinated complementary foods in the study community as an additional tool to address iron and zinc deficiency. To achieve the objective, this study addresses the following questions: i) Are families preparing and consuming fermented or germinated foods in this community? ii) Are children aged 6 to 23 months offered germinated or fermented foods? iii) What perceptions exist in the community regarding fermented or germinated foods? This information is critical to designing and implementing an intervention that promotes the use of fermented and germinated complementary foods.

## Methods

**Study site:** the study was conducted in three sub-locations of the west Kwabwai location in Homa-bay County, western Kenya in December 2019. These sub-locations are populated exclusively by members of the Luo ethnic group and selected because of the high prevalence (25%) for malnutrition (by inference iron and zinc micronutrient deficiencies). Poverty levels in the county remain a challenge and are among the highest in the country. More than three-quarters of the population survives on < 1 United States dollar/day, the World Bank's definition of extreme poverty. Subsistence farming is mostly for sorghum, potatoes, cassava, beans, and maize.

**Study design:** exploratory mixed methods research. The qualitative arm preceded the quantitative. The qualitative study explored local attitudes and understanding of fermented and germinated foods and identified native names.

**Study focus:** the study focused mainly on the knowledge, attitudes, and usage of germinated and fermented complementary foods for children 6-23 months old.

**Sample size:** focus group discussions (FGD): 67 discussants participated in Six FGDs (two in each sub-location). Focus group discussions stopped upon saturation of information. The target population in this study were caregivers of children aged between 6-23 months in Homabay County. Using the EPI info StatCalc (statistical calculator) software for cross-sectional study [22,23], an estimated that a minimum of 314 caregivers were sampled to meet a 95% confidence and a power of 80%. It was estimated that 30% of the caregivers used germinated and fermented food products as complementary foods. An additional 30 caregivers were added to cater for refusals and missing data.

**Sampling technique:** three sub-locations among five sub-locations in West Kwabwai were randomly selected for this study. Caregivers for the FGD were randomly selected from pools of individuals per sublocation that met the inclusion criteria. In each sub-location, both youth (between 18-34 years of age) and adults (35-60 years of age), married, and single caregivers were invited to the discussions.

**Survey:** using community health volunteers (CHV), children aged 6-23 months and their caregivers in each of the three sub-locations were enlisted. A population-weighted simple stratified sampling procedure was used to identify study subjects in each sub-location to ensure age and gender representation.

**Data collection tools:** a structured questionnaire was developed to collect survey data on three different themes namely: a) socioeconomic status and demographic; b) feeding practice and c) KAP of fermented and germinated foods. Questions were both closed and open-ended. A semi-structured FGD guide was used in the FGD. The discussion guide covered familiarity with germinated and fermented foods, and attitudes in the community regarding the use of germinated foods among children and adults.

**Data collection procedures:** the interviewers were trained for two days on the administration of the questionnaire including field testing of the questionnaire for clarity and comprehension in a sub-location that was not participating in the study. Changes were made to the questionnaire accordingly. Local community health workers mobilized FGD caregivers. Further screening was done on-site to make sure that caregivers met the inclusion criteria before obtaining consent. All FGDs were carried out in Dholuo, which is the language used in the study area. A moderator who had undergone training before the data collection exercise steered the discussions. However, the direction the discussion took depended on the participant's responses and areas that the moderator felt needed probing. All voice data from the FGDs were tape-recorded and later transcribed. The information was used to create a detailed reconstruction of villagers' KAP about germinated/fermented foods. The survey data was collected through a face-to-face interview with the caregiver using the structured questionnaire. Interviews were conducted in the health facility compound. Caregivers were given the opportunity to refuse to participate in the study and were assured of confidentiality.

**Statistical analysis:** statistical Package of Social Sciences (SPSS) for Windows version 25 (Inc., Chicago, IL, USA) was used for statistical analyses of quantitative data. Categorical data are presented as frequencies and percentages. Knowledge, attitude, and practice were scored by assigning points to appropriate answers and totaling their points. A cut-off determines good or poor knowledge and attitude. The chi-square test was used to identify the association between independent variables (Attitude, Knowledge, demographic, and socioeconomic variables) with the use of germinated and fermented foods (independent variable). Furthermore, binary logistic regression models were computed to test the strength of the association between the independent variable and dependent variables that showed a significant association in the bivariate analysis. Odds ratio (OR) and 95% CI for the regression parameters are reported. Statistical significance was set at p<0.05. Qualitative data analysis was done using Atlas-ti version 22. The analysis focused on the interpretation, description, and recording/writing of verbatim responses. The transcripts were first created in the Dholuo, the local language, and then translated into English. The English translation was back-translated into the Dholuo to ensure that the English and Dholuo versions carried the same meanings. The investigation team then went through the transcripts and identified emerging themes. The investigators identified suitable codes that were assigned to emerging themes from the transcripts. We uploaded the codes into the coding section in the Atlas ti software retrieved quotes from the output monitor and arranged them according to themes.

**Ethical considerations:** the Kenyatta University Ethical Review Committees (ERC) and the Institutional Review Board of the National Commission for Science, Technology and Innovation (NACOSTI approved this study protocol. The Homabay County Health and Education Departments also approved the study. The study's objectives were explained to local administrators, opinion leaders, village elders, and community members. Informed consent was sought from the study caregivers. We assured caregivers of the confidentiality of their data and erased personal identifiers before analysis.

## Results

**Sociodemographic characteristics of caregivers in FGD and survey:** sixty-seven and 314 caregivers participated in the FGD and survey, respectively. Table 1 outlines the sociodemographic characteristics of the FDG and survey caregivers. Both arms of the study had women only, with over half of them being under 35 years old. A majority of the caregivers attained the primary level of education, with 82% and 56% in the FGD and survey, respectively. In both the FGD and the survey, eight out of ten caregivers (>85%) were married, and most of them were farmers (>53%). Approximately 80% of the caregivers reported earnings of less than KES 3,000 (USD 30) per month in both the FGD and the survey.

**Table 1:** sociodemographic characteristics of caregivers in the focus group discussions and survey

Indicators	Frequency (Percentage) n=67	Frequency (%) n=314
	**FGD**	**Survey**
**Sex**		
Male	0 [0%]	0 (0%)
Female	67 (100%)	314 (100%)
**Age group**		
15-34 years	36 (53.7%)	185 (59%)
≥ 35 years	31 [46.3%]	129 (41%)
**Location**		
Got-Kojowi	22 (27.1%)	107 (34%)
Lwandawiti	21 (38.9%)	85 (27%)
Wachara	24 (34.0%)	122 (39%)
**Education level**		
≤ Primary	55 (82.1%)	176 (56%)
≥ Secondary	12 (17.9%)	138 [44%]
**Marital status**		
Married	58 (86.5%)	272 (87%)
Single	9 [13.5%]	25 (98%)
Others		17 [5%]
**Occupation**		
Farmer	37 (55.2%)	167 [53.2%]
Housewife	13 (19.4%)	49 [15.6%]
Others	17 (25.4)	98 (31.2)
Monthly income	53 (79.1%) ≤ Kes 3000	276 (88%)

### Qualitative findings

**Assessment of knowledge and awareness:** out of twelve home-made fermented foods, fermented milk and porridge were the most mentioned foods. The older caregivers identified a variety of foods compared to younger caregivers. A 39-year-old caregiver who mentioned most of the foods said, *“This dot-com generation only know potato chips and indo-meal, they will not know the foods you are asking them... I doubt that they can even cook food other than porridge.”* Most of the caregivers mentioned local brew as the only germinated food and beverage in this community. However, a few caregivers knew of germinated porridge.

**Knowledge to prepare germinated/fermented foods:** the majority of young caregivers were not able to prepare fermented foods except porridge and milk. Learning the germination and fermentation techniques was through the transfer of knowledge and skills from grandparents and their mothers. The techniques, however, varied among the caregivers. A 24-year-old caregiver said, *“I know how to ferment maize and millet porridge and of course milk...you soak the flour in water and wait for it to appear like it is boiling the following day...then prepare porridge.”* Another caregiver said, *“I usually soak the flour in little water then keep it out in the sun until the following day. It is ready for preparing porridge”* (34 years old). While an older caregiver said, *“I would keep my mixture of flour in the sun and near the cooking place that is warm at night for three to five days for me to get the good taste of porridge…it has to be sour, which is what I like”* (44 years old). A few caregivers explained how to prepare fermented foods besides porridge and milk. These foods were akeyo, fermented ugali (kuon anang´a), fermented cassava leaves, and beans. A 39-year-old caretaker of four children described how she would prepare local fermented vegetables (akeyo), *“I would boil the local vegetables until they are ready, every day I keep adding milk, boil a little, repeat this process for a week, at the end of the week, the vegetable is sour and very tasty*. Generally, caregivers thought that germination and fermentation techniques were being lost. They attributed this to modernization where girls spend most of their early childhood and youthful years in boarding schools denying them the opportunity to acquire knowledge from their grandparents as explained by a 36 years caregiver; *“in the olden days, girls learned the art of cooking from their grand-parents ...nowadays that is not the case, as young children join boarding schools when they are 10 years...” Another older caregiver concurred, “Nowadays, children are not staying with their grandmothers and their mothers have no time to prepare those foods” (39 years old)*.

**Use of germinated or fermented foods among children:** fermented or germinated foods are not commonly fed to children in this community. Only fermented porridge and milk were mentioned by the caregivers. Even so, only three caregivers could confirm ever giving their children fermented porridge. As expressed by a 35-year-old caregiver, *“My two children have always been consuming fermented porridge which we prepare for the rest of the family.” She further shared her experience,“ initially the children did not like the porridge, but now they consume just like any other member of the family”*. None of the caregivers gave their children the other fermented or germinated foods.

**Attitudes towards germinated and fermented foods:** caregivers who said they could not give their children fermented or germinated foods cited several reasons. A majority of the caregivers shared a 24-year-old caregiver's view that fermented foods caused diarrhea among children *“I cannot give my child fermented foods, including porridge, because it causes orinyacha - diarrhea in children.”* A 33-year-old caregiver added that *“fermented foods have much acid, that will make my child develop ulcers too early, and the child might be sickly throughout.”* A majority of the caregivers shared this opinion that a child's stomach is too tender to be exposed to sour foods. A 26-year´ caregiver went on to explain, *“...doctors tell ulcer patients not to take sour foods because of much acid, the foods include sour porridge, what will happen to a child who has a very delicate stomach, I don't want my child to be sick from sour porridge.” A 36 years caregiver remarked, “I see people getting drunk when they take germinated cereals made into a local brew, I cannot give my child germinated foods even porridge because that is how you introduce your child to being a drunkard in the future, and his body will get tolerant to alcohol.”* One caregiver cited the high cost of ready-made fermented porridge flour as a justification for not preparing fermented porridge. Another obstacle to consuming fermented and germinated foods was the time required to prepare them, which was deemed too long by most caregivers, particularly the younger caregivers.

**At risk of micronutrient deficiencies:** more than half of the caregivers thought their children were at risk of anemia, but they attributed it to frequent episodes of malaria in the region. All caregivers believed that anemia is a severe disease and that malaria, worm infestation, and bad eating exposed children to it. Although more than half of the caregivers knew that inadequate nutrition was a factor for anemia, the link between germination and fermentation of foods with improved nutrition was not known.

### Quantitative findings

**Knowledge about fermented and germinated foods:** Table 2 shows caregivers' knowledge about fermented and germinated foods in the study community. All 314/314 (100%) and a majority of 228/314 (73%) had heard about fermented milk, porridge, and traditional vegetables, respectively. However, only 13/314 (4%) knew about germinated porridge. Moreover, the majority (300/314, 96%) and 285 (91%) did not know how to prepare fermented milk and porridge, respectively. Only 11/314 (4%) knew how to prepare germinated porridge.

**Table 2:** percentage of carers that knew the common fermented and germinated foods in the community and how to prepare them

Common fermented food products	Heard of the food	Know how to prepare
Milk	100	96%
Maize, millet, and sorghum porridge	100	91%
Traditional vegetables (saga, cow peas leaves, jute mallow, leaves of arrowroot.	73%	18%
Beans, green grams	24%	15%
Maize meal (kuon ananga)	18%	11%
Germinated food products		
Porridge (rare) - germinated and fermented	4%	4%
Local brew - germinated and fermented	56%	6%

**Attitude towards fermented and germinated foods:** as shown in Table 3, few caregivers reported positive benefits of using fermented and germinated foods for children aged 6-23 months. In contrast, a majority of caregivers, 223/314 (71%) and 163/314 (52%), cited child developing diarrhea and child refusal to consume fermented and germinated foods as the top reasons for not utilizing the foods, respectively.

**Table 3:** caregiver's attitude towards fermented and germinated foods for children aged 6-23 months and the percentage who stated reasons for not using them

Attitude towards use of fermented and germinated foods by children	Strongly agree n (%)	Agree n (%)	Disagree n (%)	Strongly disagree n (%)	Reasons for not using the foods as complementary foods	n (%)
Improves taste for child	3 (1)	6 (2)	238 (76)	67 (21)	Diarrhea	233 (71)
Removes toxins from food	3 (1)	1 (0)	300 (96)	10 (3)	Child will develop ulcers	131 (42)
Helps child grow	10 (3)	3 (1)	278 (89)	23 (7)	Child refused	163 (52)
Food is soft for child	6 (2)	13 (4)	189 (60)	106 (34)	Child will get sick	69 (86)
Child eats more	15 (5)	14 (4)	176 (56)	109 (35)	I cannot prepare the food	98 (31)
Enhances flavor	9 (3)	10(3)	225(72)	70 (22)		
Enhances color	3 (1)	0 (0)	301 (96)	10 (3)		
Makes food last longer	7 (2)	277 (88)	5(1)	25 (8)		

**Usage of fermented and germinated foods by caregivers and children:** Table 4 shows that among the caregivers, a majority of caregivers consumed fermented milk and porridge. However, very few offered the same foods to their children aged 6-23 months, respectively.

**Table 4:** percentage of caregivers who reported utilizing fermented and germinated foods in their diets and those who use fermented and germinated foods for children aged 6-23 months

Fermented food products	Used as complementary food	Consumption by adults (caregiver)
Milk	25 (8%)	298 (95%)
Maize, millet, and sorghum porridge	9 (3%)	189 (60%)
Traditional vegetables (saga, kunde, mrenda, leaves of arrowroot)	2 (1%)	146 (46%)
Pulses (Beans, green grams)	1 (0%)	45 (14%)
Maize meal (kuon ananga)	0	31 (10%)
Germinated food products		
Porridge	0	9 (3%)
Local brew	0	31 (10%)

**Association between usage of fermented or germinated foods with the socio-demographic variables:** as shown in Table 5, the utilization of fermented and germinated complementary foods was most influenced by the caregiver's attitude, age, and level of education. Caregivers with a good attitude exhibited a significantly higher odds ratio (AOR 4.897, 95% CI: 1.616-14.844), about five times greater than the odds found in caregivers with a negative attitude. In addition, caregivers aged 35 years and older exhibited the highest odds ratio (AOR 2.7, 95% CI: 1.670-4.428), which was 2.7 times more than the odds found in caregivers aged 15-34 years. Caregivers with no education or only primary school had a significantly higher likelihood (AOR 3.344, 95% CI 1.319-8.480) of using fermented or germinated foods, compared to caregivers with a secondary and higher level of education.

**Table 5:** the relationship between use of fermented or germinated foods with key sociodemographic characteristics of the caregivers

Bivariate analysis		Binary logistic regression	
**Independent variables**	**P-value**	**Adjusted odd ratio**	**P value**
Monthly income	0.308		
Occupation	0.472		
Marital status	0.876		
Cluster	0.164		
Education level	0.013	*3.344 (1.319-8.480)	0.011
Age of caregiver	0.017	*2.7 (1.660-4.428)	0.017
Knowledge	0.324		
Attitude	0.005	*4.897 (1.616-14.844)	0.005

* Secondary and higher level of education and caregiver 15-24 years are the comparators

## Discussion

We conducted a qualitative study to explore local attitudes and understanding of fermented and germinated foods and identified native names. The quantitative research enhanced the validity of the qualitative findings.

**Knowledge of fermented and germinated foods:** this study found that a range of fermented foods were consumed in this community. Fermented milk, porridge, and local brew were the most often mentioned foods. Furthermore, a majority of the caregivers reported having consumed fermented foods at home. With the exception of local brew (Busaa), only a small proportion of caregivers had heard of or eaten germinated foods. Varieties of fermented foods are consumed throughout Africa, where household-level traditional fermentation is still part of the culture [24,25]. In Southern Africa, cereal-based mahewu and munkoyo are popular fermented products [26,27]. Togwa made from fermented sorghum, maize, millet, and maize-sorghum and traditionally fermented milk, amabere amaruranu, are common in Tanzania and Kenya, respectively [28,29]. Similarly, to our findings, fermented milk, sour porridge, and alcoholic drinks are the most popular products in Africa [24,30]. It is noteworthy that traditional fermentation processes varied among our caregivers. Whereas some caregivers soaked the cereal flour for 24 hours, others preferred a longer duration for the gruel to be sour. The lack of standardization could explain the varied opinions on the usage of fermented foods. For example, those who preferred extreme sourness in their foods preferred not to feed the foods to their children for fear that the foods would be too sour for their children's delicate digestive systems.

**Attitude towards fermented and germinated foods:** processing of foods through fermentation and germination has been shown to offer health benefits to humans. Studies have reported that fermented foods taste better and last longer without spoiling [31,32]. Similarly, the caregivers in this study reported that fermented and germinated foods tasted better and lasted longer without spoiling. Other studies have reported further benefits of fermented foods, which our study caregivers did not mention. For instance, in Nigeria, fufu, gari, and ogi have been used as preventive methods or treatments for diarrhea or dysentery and common stomach upset [33]. There is long-standing evidence that fermentation reduces the antinutrients in cereal, improving digestibility and the bioavailability of micronutrients [34]. Notably, the majority of caregivers in this study did not believe that fermented and germinated foods conferred any benefits to young children. Moreover, although a majority of the caregivers believed their children were at risk of anemia, they did not know of any association between the consumption of fermented foods and anemia. A majority of the caregivers did not approve of fermented food products for their children. The prevailing concern among caregivers was diarrhea among children after consuming fermented foods. Caregivers were also concerned that their children's digestive systems were too fragile to withstand the acidity associated with fermented foods. Contrary to the caregivers´ concerns, several studies found a reduction in diarrhea and constipation cases among children consuming fermented foods [35]. This finding may be attributed to the growth inhibition of harmful microbes in a lactic acid-fermentation environment [36-38]. Diarrheal illness among our research caregivers, especially among children, may indicate an underlying food safety issue [39] states that the initial high contamination of the raw materials and unsafe handling procedures, including storage, are to blame for episodes of diarrhea among children consuming fermented foods.

**Usage of fermented and germinated foods:** usage of fermented and germinated foods, especially among children in this community is low. However, older caregivers were more likely to use fermented and germinated foods for children. This could be explained by the generational divide evident among the study caregivers in terms of the knowledge, attitudes, and practices related to fermentation and germinated food products. Older caregivers exhibited a high level of knowledge of germinated and fermented food products. Also, they were able to describe the preparation procedures fluently as compared to the young caregivers. A positive attitude was a significant determinant of the use of fermented and germinated foods. Whereas some young caregivers did not think highly of the fermented foods in terms of taste and flavor, their older counterparts thought that the fermented foods tasted much better compared to the unfermented foods. Generally, in spite of the nutritional benefits, consumption of traditionally fermented and germinated foods has decreased [27,40]. Changing lifestyles, tastes, foreign food adoption, and time constraints are pushing the youth, particularly those in urban areas, to opt for fast foods, eroding knowledge and skills in food preparation and conservation [12]. Further, the decline in consumption of traditionally processed foods could be associated with the fact that they are considered to be for the poor [41]. When people's lifestyles improve, fermented foods and indigenous vegetables remain poor people's foods [42]. The traditional methods of knowledge transfer have also been negatively impacted by the long periods that youths are forced to be away in schools and colleges, denying their parents an opportunity to pass over the knowledge and skills they have acquired [12,43]. This could potentially explain the observed inverse relationship between the use of fermented and germinated foods and the level of education. Caregivers with secondary and tertiary education were less likely to use these foods.

## Conclusion

This study shows that, while caregivers had some knowledge and skills about germinated and fermented foods, half-truths and myths about children's consumption of fermented foods, as well as a lack of knowledge about the benefits of consuming fermented foods, could stymie an interventional project in this community. Although the community is cognizant of the risk of anemia, especially among children, the community does not understand the association between consuming fermented or germinated food products and a reduction in micronutrient deficiencies. The fear of harming children with fermented or germinated foods, especially the risk of diarrhea, needs to be adequately addressed. To reduce the risk of diarrhea associated with fermented products, standardization of practices, improved hygiene through caregiver health education, and the use of commercially regulated fermented products are required.

### What is known about this topic

- Fermentation and germination of plant-based foods reduce the concentration of anti-nutrients, hence increasing the bioavailability of iron and zinc;
- In Africa, the use of home-made fermented and germinated foods is decreasing as a result of changes in lifestyle.

### What this study adds

- The study community has an unfavorable attitude towards children's use of fermented and germinated foods;
- Diarrhea caused by fermented and germinated foods is the primary concern of the majority of caretakers, and thus a barrier to their usage in children.
